# Advanced Imaging for Congenital Left Ventricular Diverticulum in a Dog: The Role of Electrocardiosynchronous CT

**DOI:** 10.3390/ani15020280

**Published:** 2025-01-20

**Authors:** Miki Hirose, Lina Hamabe, Kazumi Shimada, Aki Takeuchi, Kazuyuki Terai, Aimi Yokoi, Ahmed Farag, Akari Hatanaka, Rio Hayashi, Katsuhiro Matsuura, Ryou Tanaka

**Affiliations:** 1 Veterinary Teaching Hospital, Tokyo University of Agriculture and Technology, Tokyo 183-8538, Japan; s212240r@st.go.tuat.ac.jp (M.H.); fv5028@go.tuat.ac.jp (A.T.); terai.kazuyuki.dvm@gmail.com (K.T.); aimi.y139@gmail.com (A.Y.); ahmedfarag9331@gmail.com (A.F.); s199337u@st.go.tuat.ac.jp (A.H.); s196832r@st.go.tuat.ac.jp (R.H.); 2 Veterinary Clinical Oncology, Tokyo University of Agriculture and Technology, Tokyo 183-8538, Japan; linahamabe@go.tuat.ac.jp; 3 Department of Small Animal Clinical Sciences, College of Veterinary Medicine, University of Florida, Gainesville, FL 32608, USA; k.matsuura.vet@gmail.com

**Keywords:** congenital heart disease, left ventricular diverticulum, ventricular septal defect, dog, echocardiography, hemodynamics, cardiac synchronous CT

## Abstract

A 2-month-old Shiba Inu was presented to an emergency veterinary clinic with decreased activity and was diagnosed with pulmonary hypertension secondary to a ventricular septal defect. The dog was referred to the TUAT Veterinary Medical Center for further evaluation. During treatment, a diverticulum was incidentally identified at the apex of the left ventricle. While the diverticulum caused no clinical symptoms, a cardiac synchronous computed tomography (CT) scan was performed to accurately assess its anatomical location and size. This case highlights the value of advanced imaging techniques in diagnosing complex congenital cardiac anomalies.

## Simple Summary

Congenital left ventricular diverticulum (CLVD) is an extremely rare congenital heart condition, with only a few cases reported in both human and veterinary medicine. It can be confused with similar conditions like left ventricular aneurysms or pseudoaneurysms, which have varying symptoms and outcomes. In this case, cardiac synchronous computed tomography (CT) successfully identified the morphology of a diverticulum in the left ventricular apex, discovered incidentally during follow-up for a ventricular septal defect, a common congenital heart disease in dogs. Cardiac synchronous CT provides detailed imaging of intracardiac and extracardiac structures throughout the cardiac cycle, making it a valuable tool for diagnosing and managing complex cardiac anomalies. This is the first veterinary report demonstrating the use of cardiac synchronous CT to evaluate CLVD.

## 1. Introduction

Congenital left ventricular diverticulum (CLVD) is a rare cardiac malformation characterized by an outpouching of the ventricular wall, typically lined by the endocardium and containing normal myocardial tissue. It may be discovered incidentally or in association with other congenital anomalies, such as abdominal wall defects or pericardial abnormalities. Although more commonly identified in humans, CLVD has been reported in veterinary cases as well, emphasizing its relevance across species (1). The symptoms and clinical implications vary widely, ranging from asymptomatic presentations to complications like arrhythmias or rupture [1,2,3]. In human medicine, asymptomatic cases are often considered benign and do not require aggressive treatment. However, potential complications include arrhythmias, thrombus formation, and rupture, necessitating careful monitoring. Differential diagnosis between CLVD and left ventricular aneurysm is essential as the two have distinct morphological and anatomical characteristics. CLVD is typically associated with a preserved myocardial wall structure, whereas aneurysms often involve myocardial thinning and scarring [4,5]. The rarity of CLVD, coupled with limited imaging data and frequent association with other congenital heart defects, makes its impact on cardiac function less well defined.

Electrocardiosynchronous computed tomography (ECG-synchronized CT) is an advanced imaging method that aligns image acquisition with the cardiac cycle, reducing motion artifacts and enabling high-resolution, artifact-free visualization of the heart’s structures [6]. It is particularly useful in assessing congenital anomalies, such as CLVD, by providing detailed morphological insights. Although ECG-synchronized CT is still limited in veterinary use, it offers a non-invasive way to evaluate complex cardiac conditions with improved diagnostic accuracy.

This case report represents the first documented use of ECG-synchronized cardiac CT for the morphological evaluation of CLVD.

## 2. Case Presentation

A 2-month-old female Shiba Inu weighing 806 g was presented to an emergency veterinary hospital with a chief complaint of anorexia and reduced activity. Physical examination revealed tachycardia with a heart rate of 180 bpm, increased respiratory effort, and cyanosis. Echocardiography revealed a Kirklin classification of membranous partial ventricular septal defect (VSD) with a right-to-left shunt and showed a flattening of the ventricular septum. The dog was hospitalized overnight in an ICU with 40% oxygen and was treated with oral sildenafil (0.5 mg/kg PO BID) and amoxicillin/clavulanate potassium (20 mg/kg PO BID). The following morning, her vital signs showed improvement, with a heart rate of 160 bpm, a respiratory rate of 56 bpm, a body temperature of 37.6 °C, and a trend toward an improved appetite.

**Figure 1:**
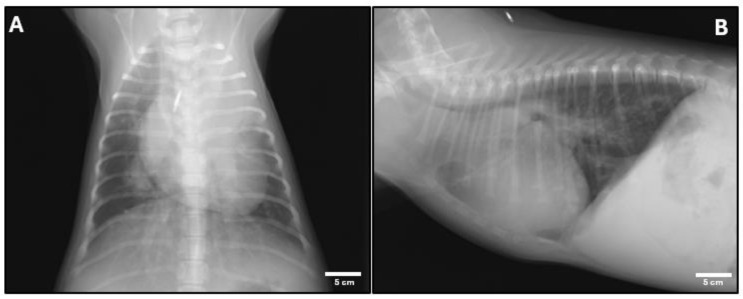
(**A**) Dorso-ventral view of the thoracic cavity showing ground-glass opacities in the right caudal lung lobe and atelectasis in the cranial left lung lobe. (**B**) Right lateral view showing an enlarged cardiac silhouette and elevation of the tracheal carina.

After her respiratory condition was stabilized, the dog was referred to the Animal Medical Center at Tokyo University of Agriculture and Technology for further evaluation. The dog exhibited increased activity and appetite, and the cyanosis had resolved; however, effortful breathing persisted. Blood tests revealed a decreased platelet count, but the coagulation profile was within normal limits. Electrocardiography revealed a right-axis deviation. Radiographic examination identified atelectasis in the left cranial lung lobe, slight opacities in the right caudal lung lobe, an enlarged cardiac silhouette, and elevation of the tracheal bifurcation (Figure 1).

Echocardiographic evaluation (LISENDO880) (Table 1) confirmed the presence of a severe membranous partial VSD measuring 6.8 mm in diameter, with a shunt blood flow velocity of 296.3 cm/s, indicative of a left-to-right shunt. Additionally, the main pulmonary artery was dilated, with a pulmonary artery-to-aorta (PA/AO) ratio of 1.59 and a pulmonary-to-systemic blood flow ratio (Qp/Qs) of 3.91 (standard value: ≤1.5). A diverticulum was also observed at the left ventricular (LV) apex in the left parasternal long-axis four-chamber view. No apparent blood flow into the diverticulum was detected at this stage, potentially due to the small size of the dog, limiting evaluation.

The dog was monitored every three weeks to manage the pulmonary hypertension associated with the VSD. On day 64, echocardiography revealed significant blood flow between the diverticulum and the LV. Consequently, cardiac synchronous CT was performed on day 128 to evaluate the anatomical structure and hemodynamic significance of the diverticulum. The CT images confirmed a direct connection between the diverticulum and the LV, indicating active blood flow between these structures.

## 3. Echocardiography

**Figure 2:**
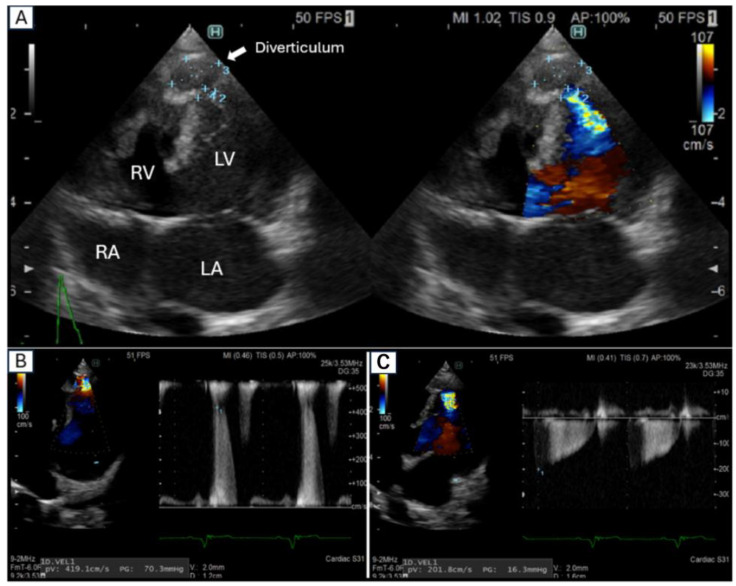
Echocardiographic assessment of left ventricular diverticulum. (**A**) Left ventricular diverticulum observed in the left apical four-chamber view. (**B**) Pulsed-wave Doppler analysis of blood flow at the diverticulum opening during systole. (**C**) Pulsed-wave Doppler analysis of blood flow at the diverticulum opening during diastole. Abbreviations: RV (right ventricle); RA (right atrium); LV (left ventricle); LA (left atrium).

The left ventricular (LV) diverticulum was identified using echocardiography, measuring 10.2 × 7.6 mm, with a 3.6 mm opening connecting it to the LV chamber (Figure 2A). Blood flow dynamics demonstrated bidirectional flow through the diverticulum: during diastole, blood flowed from the diverticulum into the LV at a velocity of 201.8 cm/s, while during systole, blood was ejected from the LV into the diverticulum at a velocity of 419.1 cm/s (Figure 2B). The assessment of myocardial wall motion was enhanced using 2D speckle-tracking echocardiography (2D-STE), which confirmed the asynchronous movement of the diverticulum wall. While asynchronous motion could be visually identified with B-mode imaging, 2D-STE provided quantitative strain analysis to characterize the mechanical properties of the myocardium. The global longitudinal strain (GLS) of the LV was measured at −18.7%, indicating preserved systolic function. Interestingly, the apex of the interventricular septum (ApS) exhibited a unique double contraction per cardiac cycle, potentially contributing to the observed asynchronous motion. Despite the presence of the diverticulum, no arrhythmias or evidence of thrombus formation within the LV apex were observed. The use of strain imaging was pivotal in this case, offering insights into the functional significance of the diverticulum, which would not have been feasible with visual assessment alone.

## 4. Cardiac Synchronous Computed Tomography

**Figure 3:**
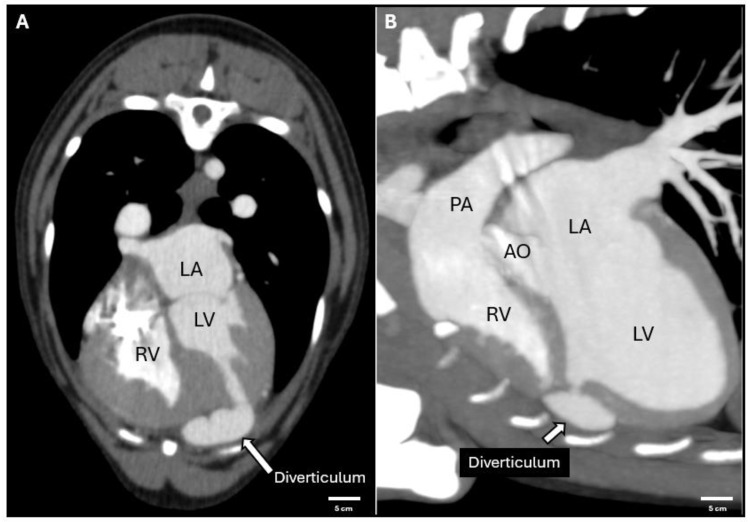
Cardiac synchronous CT imaging of the left ventricular diverticulum. (**A**) Transverse plane demonstrating the anatomical location of the diverticulum adjacent to the sternum (transverse section of the fourth vertebral body). (**B**) Sagittal plane illustrating the absence of myocardial tissue surrounding the diverticulum and lack of communication with the right ventricle. Abbreviations: LV (left ventricle); RV (right ventricle); LA (left atrium); AO (aorta); PA (pulmonary artery).

Cardiac synchronous computed tomography (CT) was performed under general anesthesia, revealing significant findings concerning the left ventricular diverticulum. The diverticulum was identified as a thin-walled structure located adjacent to the sternum, with no visible myocardial tissue surrounding the diverticulum. The absence of myocardial components was consistently confirmed across transverse, sagittal, and coronal planes, ensuring comprehensive anatomical evaluation. Contrast-enhanced CT studies showed no evidence of communication between the diverticulum and the right ventricular chamber, effectively ruling out any significant right ventricular traffic (Figure 3). Additionally, the diverticulum appeared as a pouch-like extension of the left ventricular wall, exhibiting clear boundaries without signs of calcification or thrombus formation. The structure’s proximity to the sternum was emphasized in 3D reconstructions, aiding in spatial orientation.

**Figure 4:**
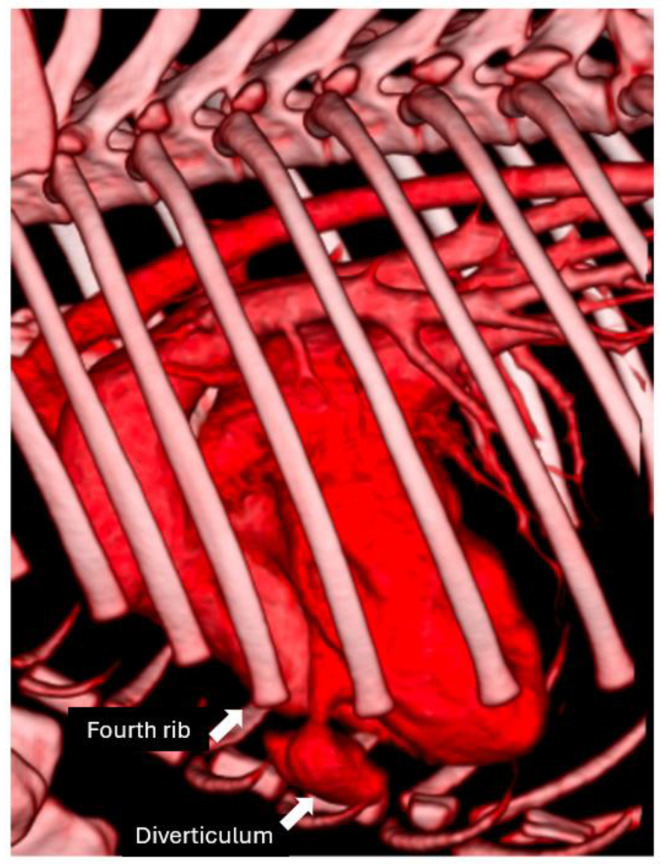
Three-dimensional reconstruction of cardiac synchronous CT. The 3D image depicts the position of the diverticulum relative to the sternum and ribs, emphasizing its dynamic changes during the cardiac cycle.

Dynamic imaging through electrocardiosynchronous CT revealed the diverticulum’s functional characteristics (Figure 4). During systole, the diverticulum expanded, demonstrating asynchronous behavior with the left ventricular contraction. Conversely, during diastole, it exhibited contraction. However, the precise contractile capability of the diverticulum remained indeterminate, as the motion could be attributed to either intrinsic muscular activity or passive mechanical forces transmitted from the left ventricle. Therefore, the expansion of the diverticulum cavity during systole may be a result of increased pressure and volume during ventricular blood ejection. This dynamic evaluation underscores the unique behavior of the diverticulum and its potential functional implications.

## 5. Discussion

The observed reduction in activity in this patient is a well-recognized symptom associated with pulmonary hypertension (PH) secondary to a VSD. However, no clinical symptoms indicative of CLVD were noted. CLVD is a rare cardiac malformation, reported in approximately 0.1% of all congenital heart diseases in humans [5]. In veterinary medicine, only one prior case of left ventricular diverticulum has been reported as an incidental finding during autopsy [1]. To the best of our knowledge, this is the first reported case of CLVD in a dog with a comprehensive evaluation using antemortem diagnostic imaging.

CLVD has been documented in various locations, including in the left ventricular apex, the aortic origin, and the right ventricle. It is histologically classified into muscular and fibrous forms. The muscular type, often connected to the left ventricle (LV) via a short passage, is believed to contract independently, although some studies suggest its synchronous movement with the LV [7,8]. Asymptomatic cases generally do not require intervention, but surgical management may be necessary in cases with a risk of rupture or complications such as arrhythmias [9,10,11]. Isolated and asymptomatic CLVD typically has a favorable prognosis [12].

In human medicine, CLVD is associated with midline endocardial defects and Cantrell’s syndrome [13,14]. In this case, the patient was initially diagnosed and treated for PH associated with a VSD, and the presence of CLVD was later identified.

Global longitudinal strain (GLS) has emerged as a reliable parameter for evaluating myocardial deformation, gaining traction in both human and veterinary cardiology. In this patient, the GLS of the LV was measured at −18.7%, a value within the normal range for dogs. This finding indicates preserved systolic function despite the presence of the diverticulum. Prior studies have emphasized the sensitivity of GLS in detecting subtle myocardial dysfunction before ejection fraction changes become apparent [15,16,17]. Interestingly, the GLS value in this case provides critical insights into the mechanical impact of the diverticulum. Despite the asynchronous movement of the diverticulum, as demonstrated by 2D-STE, the global strain pattern of the LV remained uncompromised. This suggests that the diverticulum had a minimal effect on ventricular compliance and contractility, likely due to its small size and limited biomechanical influence.

Additionally, the unique observation of dual contractions at the apex of the interventricular septum may reflect a localized compensatory mechanism aimed at preserving effective ventricular function. Similar phenomena have been reported in other congenital anomalies, where segmental biomechanical alterations help maintain global function [18]. The absence of arrhythmias or thrombi further underscores the clinical stability of the diverticulum, although the potential for future complications, such as rupture or thromboembolism, necessitates regular monitoring.

This case underscores the importance of integrating advanced imaging modalities in diagnosing and evaluating congenital cardiac anomalies. While B-mode imaging identified asynchronous motion, strain imaging quantified the degree of myocardial deformation, offering a more detailed assessment of both regional and global ventricular function. The preserved GLS highlights the utility of 2D-STE in cases where structural anomalies coexist with seemingly normal global function. During the initial evaluation, the diverticulum was misdiagnosed as pericardial effusion. Subsequent detailed examination during vector flow mapping (VFM) at follow-up revealed the presence of the diverticulum. Careful evaluation using color Doppler and pulsed-wave Doppler confirmed blood flow communication between the LV and the diverticulum, while asynchronous motion was verified using strain analysis. Furthermore, cardiac synchronous computed tomography (CT) provided valuable insights into the gross anatomy of the diverticulum, confirming the absence of additional congenital abnormalities apart from the VSD and diverticulum.

Histologically, CLVD comprises three myocardial layers [19,20]. Although histopathology was not performed, an aneurysm cannot be ruled out, but in this case, the clinical course, tests performed, and morphology strongly suggest CLVD. Isolated CLVD is rarely a direct cause of heart failure and often remains undiagnosed. The development of advanced imaging technologies is likely to increase the detection rate of such anomalies in both human and veterinary medicine [19,21,22].

This report provides a detailed account of CLVD in a dog, emphasizing the importance of advanced diagnostic imaging in detecting and characterizing this rare anomaly. While the diverticulum’s impact on global myocardial function was minimal in this case, its potential for future complications warrants continued surveillance. Further studies are needed to better understand the pathophysiology, prognosis, and management of CLVD in veterinary patients.

## 6. Conclusions

CLVD is an exceptionally rare cardiac anomaly, with limited reports in both human and veterinary medicine. This case represents the first documented instance of suspected CLVD confirmed antemortem in a dog. Through the integration of advanced imaging techniques, including echocardiography to evaluate hemodynamic function and gated cardiac CT to define morphological and anatomical characteristics, this report provides valuable insights into the diagnosis and assessment of CLVD in canine patients. We hope that this case will serve as a foundation for advancing the understanding of CLVD and its clinical implications in veterinary medicine.

## Figures and Tables

**Table 1:** Results of echocardiographic examinations performed at day 18 and day 128.

Parameters	Day 18	Day 128
LVIDd (mm)	18.4	23.0
FS (%)	48.3	51.6
RVOTv (cm/s)	214.1	208.1
LVOTv (cm/s)	107.0	100.6
Ev (cm/s)	131.1	83.2
E/A	1.03	0.87
s’sep (cm/s)	9.0	7.9
e’sep (cm/s)	6.8	4.5
E/e’sep	19.38	16.71
s’lat (cm/s)	7.4	12.9
e’lat (cm/s)	11.1	7.1
E/e’ lat	11.77	10.64
MRv	trivial	trivial
VSDv (cm/s)	296.3	235.0
VSD diameter (mm)	6.8	7.2
PRv (cm/s)	trivial	427.0
PA/AO	1.59	1.60
Qp/Qs	3.91	4.97

LVIDd: left ventricular end-diastolic dimension; FS: fractional shortening; RVOTv: right ventricular outflow tract velocity; LVOTv: left ventricular outflow tract velocity; Ev: E wave velocity; E/A: E-wave-to-A-wave ratio; s’sep: systolic peak velocity of the septal mitral annulus; e’sep: early diastolic velocity of the septal mitral annulus; MRv: mitral regurgitation velocity; VSDv: ventricular septal defect velocity; PRv: pulmonary valve regurgitation velocity; PA/AO: pulmonary-to-aortic ratio; Qp/Qs: pulmonary-flow-to-systemic-flow ratio.

## Data Availability

The original contributions presented in the study are included in the article; further inquiries can be directed to the corresponding author.
